# A statistical model for multidimensional irreversible electroporation cell death in tissue

**DOI:** 10.1186/1475-925X-9-13

**Published:** 2010-02-26

**Authors:** Alex Golberg, Boris Rubinsky

**Affiliations:** 1 Center for Bioengineering in the Service of Humanity and Society, School of Computer Science and Engineering, Hebrew University of Jerusalem, Givat Ram, Jerusalem 91904, Israel; 2 Department of Mechanical Engineering, Graduate Program in Biophysics, University of California at Berkeley, Berkeley CA 84720, USA

## Abstract

**Background:**

Irreversible electroporation (IRE) is a minimally invasive tissue ablation technique which utilizes electric pulses delivered by electrodes to a targeted area of tissue to produce high amplitude electric fields, thus inducing irreversible damage to the cell membrane lipid bilayer. An important application of this technique is for cancer tissue ablation. Mathematical modelling is considered important in IRE treatment planning. In the past, IRE mathematical modelling used a deterministic single value for the amplitude of the electric field required for causing cell death. However, tissue, particularly cancerous tissue, is comprised of a population of different cells of different sizes and orientations, which in conventional IRE are exposed to complex electric fields; therefore, using a deterministic single value is overly simplistic.

**Methods:**

We introduce and describe a new methodology for evaluating IRE induced cell death in tissue. Our approach employs a statistical Peleg-Fermi model to correlate probability of cell death in heterogeneous tissue to the parameters of electroporation pulses such as the number of pulses, electric field amplitude and pulse length. For treatment planning, the Peleg-Fermi model is combined with a numerical solution of the multidimensional electric field equation cast in a dimensionless form. This is the first time in which this concept is used for evaluating IRE cell death in multidimensional situations.

**Results:**

We illustrate the methodology using data reported in literature for prostate cancer cell death by IRE. We show how to fit this data to a Fermi function in order to calculate the critical statistic parameters. To illustrate the use of the methodology, we simulated 2-D irreversible electroporation protocols and produced 2-D maps of the statistical distribution of cell death in the treated region. These plots were compared to plots produced using a deterministic model of cell death by IRE and the differences were noted.

**Conclusions:**

In this work we introduce a new methodology for evaluation of tissue ablation by IRE using statistical models of cell death. We believe that the use of a statistical model rather than a deterministic model for IRE cell death will improve the accuracy of treatment planning for cancer treatment with IRE.

## Background

Electroporation is the physical phenomenon in which the cell membrane becomes permeabilized when certain electric fields are applied across the cell [1]. When cell membrane permeability increase is only temporary and the resealing happens in the next step, reversible electroporation has occurred [2-8]. Reversible electroporation has important applications in chemical treatment of tissues for drug delivery and gene therapy [9-11] If permeability increase is sufficiently long to disrupt intracellular homeostasis, irreversible electroporation has occurred and as a consequence the cell dies [12]. Until recently, the main practical application of irreversible electroporation was microbial inactivation in the food industry [13-15]. A summary of much of the current information on the use of IRE in the food industry can be found in a recent book on this topic [15]. The use of irreversible electroporation in a non thermal mode for tissue ablation in the body *in vivo *is a new minimally invasive molecular selective surgical technique [16-21]. Tissue electroporation utilizes electrodes brought into contact with tissues in the body to deliver electric pulses, which in turn induce electroporation in a desired volume of tissue [22,23]. Non-thermal irreversible electroporation (NTIRE) is electroporation delivered in such a way that the Joule heating induced temperature elevation in tissue only reaches levels that are not harmful[24]. Therefore, only the cell membrane in the treated area is affected while other molecular structures in the tissue are spared, effectively making NTIRE molecular surgery[23,25]. One application of NTIRE is the treatment of cancerous tumors [16,17,20,23]. In a typical procedure, electrodes are inserted around the tumor and pulses of specific amplitude and frequency are applied in the hope that they will affect the entire area of the tumor and cause complete cell death [16,17,20,23]. Treatment planning is important for NTIRE treatment success. In the past, mathematical studies on electroporation in tissue used a deterministic model to evaluate the electroporation events, i.e. it was assumed that the event of electroporation is associated with a single value of local electric field current and heat distribution during pulse application[17,21,24-33]. Particular attention was paid to the electrode confirmation optimization [34,35] and the impact of tissue histology [36]. Nevertheless, assuming a deterministic effect of electroporation parameters is correct only when the cell population is homogeneous and uniform. In malignant tissues the cell population is at different stages of development and is therefore not homogeneous. It has been known in the field of irreversible electroporation since the 1960's that in a population of aging cells there is a statistical distribution which correlates cell survival to electroporation parameters [37,38]. The outcome of the application of electric pulses across cells depends on many parameters. These include field amplitude, polarity, number of electric pulses, shape of pulses, length of pulse, interval between pulses, and environmental temperature. Particularly relevant to tissue are the additional parameters of cell type, morphology, age and size [2-8,26,37,38]. All these parameters determine if the cell membrane will undergo reversible electroporation, irreversible electroporation or no electroporation at all. When treating cancer cells with NITRE, it is obviously important to deliver the electric pulses such that the electric conditions that destroy cells are achieved throughout the entire volume of targeted undesirable tissue. The use of NTIRE for tissue ablation is complicated by the fact that the electric fields which occur in the treated tissue are complex and vary in space as a function of distance from the electrodes, tumor and electrode geometry e.g [17,25]. Therefore, there is evident need for a mathematical methodology of treatment planning which will ensure that the entire volume of undesirable tissue undergoes electric conditions that destroy all the cells.

The food industry, from which some of the first fundamental studies on IRE emerged [37,38] has long recognized that electroporation is a statistical event in a heterogeneous population of cells. In food processing, it is important to completely destroy undesirable cells; as is in treatment of cancer. Therefore, statistical models of cell destruction by irreversible electroporation have been developed in the food industry for processing planning. Our goal in this study is to show how these models can be used in treatment planning for ablation of cancer cells in tissue.

The first mathematical models to describe pulsed electric field induced cell death employed a first order inactivation kinetics model and are given in equation (1), [39](1)

Where S is the survival ratio, k is the kinetic constant which depends of pulse strength and t is the total treatment time.

However, experimental studies show that cell death by pulsed electric fields depends on more parameters than those included in a first order kinetic model. Hülsheger and Niemann proposed a model which is different from first order inactivated models and incorporates more of the relevant pulsed electric field parameters, Equation (2), [40]:(2)

Where b_e _is a regression constant, which is bacteria and medium type dependent. E is the applied field and E_c _is a cell size and pulse length dependent parameter, obtained by extrapolation to 100% survivals. Further model development [14,41,42] have lead to the model in Equation 3, which also includes brings the pulse length as a critical parameter in electric pulse field induced cell death:(3)

Where t_c _and E_c _are microorganism and medium type dependent, E is the applied field and t is the treatment time.

Additional models were developed which take into account the fact that the treated microorganisms population is not homogeneous, hence each individual cell has its own resistance to the applied treatment. Assuming a natural distribution among cells, the survival curve can be described by a distribution function[43-45].

Peleg [46]proposed an inactivation model, Equation 4, based on Fermi function:(4)

Where, Ec(n) is the field at which 50% of a population of cells are dead and A(n) are function of the number of pulses, n.

Recently, a Weibull distribution, function has been shown to describe effectively several microbial inactivation curves, Equation 5, [44,45]:(5)

Where n(E) and b(E) are constants and depend on microbial and media type and treatment parameters (electric field and treatment time).

Several additional models have been reported in the literature [47-49]. San Martin et al [50] and Alvarez et al [51] made a comparison study of several proposed statistical models.

The statistical mathematical models used in the food industry deal with one dimensional electric field. These models have practical value in the food industry because the majority of the geometrical configurations in which IRE is used in that industry are one-dimensional. However, when irreversible electroporation is used for medical treatment the electric fields that develop in the treated tissue they are seldom one dimensional[17]. In developing NTIRE mathematical models for medicine it would be beneficial to have a methodology that could predict the outcome of a particular electroporation treatment in tissues made of a variety of cells that experience multidimensional and complex electric fields at complex electroporation protocols.

The goal of this study is to introduce such a methodology, which will lead to the treatment planning according to parameters we previously discussed. Specifically, we suggest combining a mathematical model that calculates the multidimensional electric field in tissue with a statistical and empirical model that predicts cellular damage as a function of the local and temporal values of electric fields and the electroporation protocols. Mathematical models that calculate the multi-dimensional electric fields which occur during tissue electroporation through the solution of the electric field equation have been used successfully in the past for electroporation analysis and research [22,52] as well as for treatment planning in NTIRE [17,20,53]. In the past these mathematical models of electric fields were combined with a deterministic single valued evaluation of the electric field that affects cell viability and the results were expressed as a demarcation line which separates between cells that were electroporated and those that are not. There has been no methodology introduced, up to our knowledge, which evaluates the statistical distribution of electroporated cells. Here we propose a second step after the electric field calculations which consists of inserting the calculated local value of the electric fields into a statistical empirical model of the type derived in the food industry for estimate of local cell damage. This analysis should produce a map of tissue damage in the treated region for a certain electroporation protocol which is the goal of treatment planning. We anticipated that the major difference in the outcome of the analysis between the methodologies proposed in this study and the mathematical methodology used in the past is the occurrence of a domain in which there will be a transition between electroporated and non-electroporated tissue, rather than a discrete demarcation line. Knowing this transition zone is obviously important in treatment of cancer.

This study describes this mathematical model of electroporation in tissue. Since we want to introduce a general methodology, we will employ dimensionless analysis - which is basic in fundamental engineering analysis. To illustrate the method we will use a Peleg-Fermi type statistical model [46]. Because there is no good experimental data in the literature for IRE in tissue and to nevertheless focus ideas we use and extrapolate limited experimental data obtained for DU 145 prostate cancer cells in a previously published work, based on *in vivo *experiments, by Canatella et al[54]. The experimental parameters in this specific study. which included field strength from 0.1 to 3.3 kV/cm, pulse length 50 *μ*sec -20 ms, number of pulses 1-10 [41], fall to the range of parameters used in vivo studies for the successful irreversible electroporation [16,20,22,53]; therefore, we applied these results for demonstration in the current 2D treatment planning model application. In the investigated electroporation study the pulse lengths were significantly longer than the cell membrane charging time which is about 1 *μ*sec [55] and thus a steady state DC analyses can be implemented. Obviously, for this method to become practical much experimental research is needed to obtain statistical data for cells in tissue.

## Methods

To develop the methodology we will employ a statistical empirical model of cell damage by electroporation based on the Peleg-Fermi formulation[46]. The reason for choosing this model over others is related to recent findings in the field of tissue NTIRE. These findings show that the number of pulses is an important treatment parameter[16,26,56]. We chose to use the Peleg-Fermi model since it directly incorporates the dependence of cell death on pulse number and field strength for the given pulse length. Other models, for instance, Weibull function parameters do not incorporate directly the pulse number and pulse length as basic parameters and include only the effect of field amplitude and total treatment time. Obviously the other models can be also used and it is quite likely that new statistical models will be developed in the future for treatment of tissue; however, this study should be viewed primarily as a first attempt at introducing statistical modeling in the analysis of tissue electroporation.

Peleg [46] depicts the dependence of the survival ratio S (S = N/N_o _or the ratio of living cell count after IRE treatment (N) and before IRE treatment (N_o_)) on the electric field that cells experience, E [V/m] and number of pulses, n, for various electroporation protocols.

The model is based on the Fermi equation of the form described in Equation 4.

The equation incorporates Ec(n) whose typical behavior is(6)

Where Eco is the intersect of the curve with the y-axis and is cell type and pulse type specific, n, is the number of pulses and k1 is cell type and pulse type specific. The pulse type specificity relates to all the other parameters of electroporation that are not included explicitly in the equation (i.e. shape of pulse, length of pulse, interval between pulses).

The equation for A(n), whose typical behavior is,(7)

The electric field during the electroporative pulses application is obtained from the solution of the Equation 8,(8)

where, *σ *[S] is the local conductivity and *ϕ*[V] is the local potential

To determine the electric potential in the analyzed region Equation (8) is solved subject to the electroporation boundary condition which are:(9)

where Σ_1_, Σ_2 _are the geometrical locations of the electroporation electrode boundaries.

Boundary conditions that do not relate to the electrodes are handled in a standard way, as insulating boundaries. A typical example will be shown later in the results section.

Since we introduce here a general methodology we will employ dimensionless analysis, as commonly done in engineering analysis. We assume that the typical dimension of this problem is the distance L [m], between the centers of gravity of the two electroporation electrodes. We will non-dimensionalize space variables with respect to the dimension, L, and electric field quantities with respect to Eco which is a typical quantity with units of electric field and dependent on the tissue type and electroporation protocol. Specifically:(11)

The dimensionless form of Equations (4) and (6-11) becomes,(12)

We anticipate that mathematical modeling of IRE will be performed the following way. The experimental data, gathered in preliminary experiments with tissues, will be cast in a statistical model of cell death as a function of various electroporation parameters rather than a deterministic model. It is quite possible that the experimental studies will reveal other parameters of importance for the statistical model; for instance, the effect of the variable polarity, anisotropic properties in relation to the electric fields, heterogeneity to mention a few. From the data gathered in the food industry we have little doubt that in tissue the cell electroporation as a function of electroporation parameters will have a statistical distribution rather than be deterministic. Then the Laplace equation is solved for the particular geometry and electroporation protocol and the statistical model can be used as a survival look-up table with the calculated local electric field to determine the transition region to complete cell death. It should be emphasized that in other tissue ablation techniques such as cryosurgery and thermal ablation this statistically affected transition region has become an important consideration in treatment planning.

## Results and Discussion

The goal of this part of the study is to illustrate the methodology with an example. Since there is no experimental statistical data available for tissues we decided to illustrate the concept using some limited data available from experiments with prostate DU 145 cancer cells in the work by Canatella et al[54], which we extrapolate. The goal of this study was to introduce the idea that electroporation effects on tissue should be analyzed as a statistical, probabilistic event rather than as a deterministic event. Tissues are obviously heterogeneous at the microscopic and macroscopic scale and often anisotropic. Others and we have published, studies on the effects of tissue heterogeneity on tissue electroporation and it is substantial [27,30,36,57-60]. However, in order to single out the effect of a statistical distribution of electroporation events on the outcome of electroporation, we chose to model the tissue as homogeneous. This approach to the analysis of a newly examined phenomenon is obviously quite standard [22,33].

We could have used data from experiments with micro-organisms from the food industry or just simple parametric studies; however, we thought that although limited, the prostate cancer cell data is somewhat more relevant. Obviously future experimental studies on tissues are needed in this field.

The data of Canatella et al [54]gives the percentage cell survival as a function of applied field intensity for 1, 2, 4 and 10 pulses with pulse lengths of 50 *μ*sec, 100 *μ*sec, 1 msec and 10 msec.

**Figure 1:**
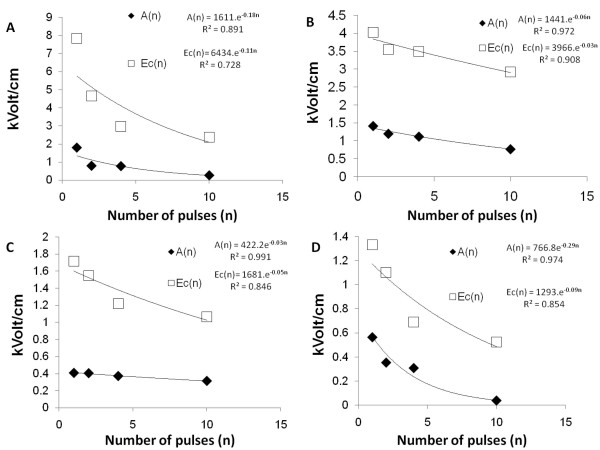
**Dependence of Ec and A on the number of pulses as developed from the work of Canatella et al **[54]. A. 50 *μ*sec pulse lenth. B.100 *μ*sec pulse length, C. 1 msec pulse length and D. 10 msec pulse length.

We have curve fitted the data of Canatella et al. [54] to the Fermi type model of Peleg, Equation 1 [46], The curve fitted parameters Ec and A as a function of n were calculated from the experimental data and are shown in Figures 1A to 1D.

**Figure 2:**
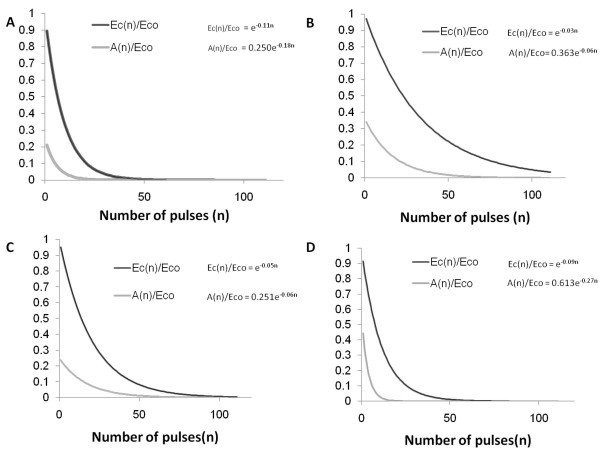
**Dependance of Ec and A on the number of applied pulses, normalized to Eco**. A. 50 *μ*sec pulse lenth. B.100 *μ*sec pulse length, C. 1 msec pulse length and D. 10 msec pulse length.

From the plots in Figures 1A to 1D we extrapolated to n = 0 to obtain the values of Eco and Ao for each electroporation protocol. The plots in Figures 1A to 1D were non-dimensionalized as in Equations 16 and 17 and further extrapolated to larger number of pulses than in the experiments of Canatlela et al[54]. These dimensionless representations are shown in Figures (2A, B, C and 2D). It should be obvious that what we show is a general methodology and the particular use of the Canatella et al[54] data is to have some basis grounded on experimentation for the description of the methodology.

We will further illustrate the methodology by analyzing a configuration that is typical to the NTIRE experiments described previously[61]. Specifically, in those experiments two long 1 mm diameter cylindrical electrodes are placed at a separation of 1 cm between them in a parallel configuration. This situation is primarily two dimensional. For simplicity we will assume that the tissue is isotropic (although the method is obviously not restricted to these conditions) with *σ *= 0.42 S/m[62].

**Figure 3:**
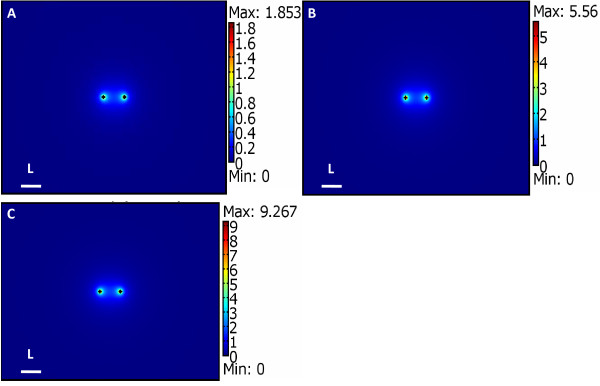
**Dimensionless electric field distribution solution in the treated tissue for A. C = 0.5, B. C = 1.5 C. C = 2.5**.

**Figure 4:**
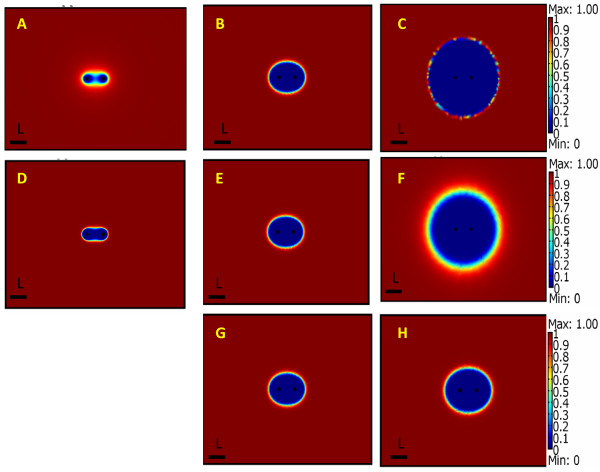
**Viability plots for IRE in prostate tissue in 2D for different electroporation protocols that have various number of pulses (n), voltages on the electrodes C, and pulse length, (t)**. A. n = 10 C = 1.5 t = 100 *μ*sec B. n = 50 C = 1.5 t = 100 *μ*sec C. n = 100 C = 1.5 t = 100 *μ*sec. D. n = 50 pulses, C = 0.5, t = 100 *μ*sec E. n = 50 pulses c = 1.5 t = 100 *μ*sec F. n = 50 pulses c = 2.5 t = 100 *μ*sec. G. n = 50 C = 1.5 t = 100 *μ*sec H. n = 50 C = 1.5 t = 1 msec.

The electric field equation is solved using the finite element method with Comsol Multiphysics (version 3.4). The paradigm of the analysis is as follows. The field equation is solved for prescribed voltage boundary conditions on the electrodes and insulating boundary conditions on the outer edges of the domain, and then the curves in Figure 2 are used to evaluate the cell survival for each value of the local field and the appropriate number of pulses and electroporation protocols. In a typical parametric treatment study we have varied the C values (dimensionless voltage on the electrodes) and treatment parameters (number of pulses and length of pulses) and plotted from the electric field data a spatial depiction of the cell survival. The calculated dimensionless field distribution in the tissue is given in Figures 3(A-C) The cell survival 2D plots are shown in Figures 4(A-H).

Figures 4(A-H) show the distribution of cells which survive IRE in relation to the location of the electroporation electrodes for various electroporation protocols. The depiction of the cell damage is obtained from the calculation of the electric fields and the use of the Peleg-Fermi type empirical data. The most important aspect of our findings is that around the treated tissue there is a rim of tissue in which the NTIRE caused damage is partial. The existence and the extent of regions in which only part of the cells are ablated cannot be determined from the deterministic cell death models which have been used before The shape of the treated region is obviously a function of the electrical parameters and the geometry of the probes. From the results it is evident that the damaged region increases as a function of applied voltage, pulse number and pulse length. Both regions of the sub-lethal injured and totally inactivated cells are changing as a function of the applied protocol. The general pattern is interesting: larger numbers of pulses increase the region in which there is complete cell death (blue color) while large field amplitude and longer pulse length increase both the region in which there is complete cell death as well as the transition region of partial cell injury (Figures 4(A-C)). These findings further illustrate the importance of using a statistical distribution model for a precise analysis of the effects of NTIRE. The geometrical form of the treated area changes its shape with the treatment parameters in a form similar to that observed in other studies [33].

In this study we introduce a methodology for evaluating cell death in a volume of tissue treated by IRE using a statistical cell death model rather than the deterministic model for cell death used in the past.

The examples shown in this study illustrate the methodology for mathematical analysis of IRE for multidimensional electroporation protocols from fundamental information on the empirical, statistical relation between cell survival and electroporation protocols in experiments and mathematical solution of the field equation. For a desired region of tissue ablation it is possible to employ this methodology for choosing the desirable electric pulse protocol in terms of pulse amplitude, length, number of pulses and intervals between the pulses. Because non-thermal irreversible electroporation also requires pulses that do not produce thermal damage future studies may also require solving this model of electric fields together with thermal models dealing with temperature distributions as well as thermal damage. While shown for irreversible electroporation this mode of analysis could be employed in a similar form with experimental curves for reversible electroporation. Obviously this is a theoretical study whose goal it is to propose a statistical model for IRE mathematical modeling. It should be empathized that the data used in this work is for illustration purposes only and real curves and parameters should be developed for each specific case. We performed the simulations based on two assumptions. First, we extrapolated data from in vitro experiment performed by Canatella et al. [54] to an in vivo situation in tissue, second we used the Peleg-Fermi model to extrapolate the effect of electric field delivered at a much larger number of pulses than was reported by Canatella et al. [54]. Eventually, in order to use the theoretical methodology introduced in this work in clinical applications experimental studies need to be performed to develop real values for statistical analysis.

The results that were obtained show that when a statistical model is used to predict cell destruction by IRE there is a transition zone between complete cell destruction and complete cell survival. In contrast, previous mathematical models of IRE which employed deterministic models show a sharp transition line. Obviously, knowing precisely the extent of complete tissue ablation is important in treatment of cancer. The mode of analysis and treatment planning design presented in this study may become important in attempts to optimize the use of NTIRE in treatment of cancer.

## Conclusion

This study has introduced a new mathematical methodology for analysis of tissue ablation by irreversible electroporation using statistical models of cell death. The methodology was illustrated using data derived from single cell studies. Much experimental work remains to obtain similar data for cells in tissue. However, once the experimental data becomes available, the use of a statistical model rather than a deterministic model for IRE cell death will improve the accuracy of treatment planning for cancer treatment with IRE.

## Competing interests

The authors declare that they have no competing interests.

## Authors' contributions

AG performed data collection modeling and drafted the manuscript. BR conceived of the study and drafted the manuscript. All authors read and approved the final manuscript.
